# Sirtuin 1 (SIRT1) Activation Mediates Sildenafil Induced Delayed Cardioprotection against Ischemia-Reperfusion Injury in Mice

**DOI:** 10.1371/journal.pone.0086977

**Published:** 2014-01-22

**Authors:** Mona Shalwala, Shu-Guang Zhu, Anindita Das, Fadi N. Salloum, Lei Xi, Rakesh C. Kukreja

**Affiliations:** 1 VCU Pauley Heart Center, Division of Cardiology, Department of Internal Medicine, Virginia Commonwealth University, Richmond, Virginia, United States of America; 2 Touro College of Osteopathic Medicine, New York, New York, United States of America; 3 Department of Cardiothoracic Surgery, The First Affiliated Hospital of Guangdong Pharmaceutical University, Guangzhou, China; University of Central Florida, United States of America

## Abstract

**Background:**

It has been well documented that phosphodiesterase-5 inhibitor, sildenafil (SIL) protects against myocardial ischemia/reperfusion (I-R) injury. SIRT1 is part of the class III Sirtuin family of histone deacetylases that deacetylates proteins involved in cellular stress response including those related to I-R injury.

**Objective/Hypothesis:**

We tested the hypothesis that SIL-induced cardioprotection may be mediated through activation of SIRT1.

**Methods:**

Adult male ICR mice were treated with SIL (0.7 mg/kg, *i.p.*), Resveratrol (RSV, 5 mg/kg, a putative activator of SIRT1 used as the positive control), or saline (0.2 mL). The hearts were harvested 24 hours later and homogenized for SIRT1 activity analysis.

**Results:**

Both SIL- and RSV-treated mice had increased cardiac SIRT1 activity (P<0.001) as compared to the saline-treated controls 24 hours after drug treatment. In isolated ventricular cardiomyocytes, pretreatment with SIL (1 µM) or RSV (1 µM) for one hour *in vitro* also upregulated SIRT1 activity (P<0.05). We further examined the causative relationship between SIRT1 activation and SIL-induced late cardioprotection. Pretreatment with SIL (or RSV) 24 hours prior to 30 min ischemia and 24 hours of reperfusion significantly reduced infarct size, which was associated with a significant increase in SIRT1 activity (P<0.05). Moreover, sirtinol (a SIRT1 inhibitor, 5 mg/kg, *i.p.*) given 30 min before I-R blunted the infarct-limiting effect of SIL and RSV (P<0.001).

**Conclusion:**

Our study shows that activation of SIRT1 following SIL treatment plays an essential role in mediating the SIL-induced cardioprotection against I-R injury. This newly identified SIRT1-activating property of SIL may have enormous therapeutic implications.

## Introduction

Acute myocardial infarction (AMI) is a cardiovascular disease that affects more than 1.5 million people in the United States annually and is a major cause of mortality and morbidity worldwide. In order to combat this serious health problem, there have been extensive investigations during the past four decades in searching for cardioprotective drugs that can be safely implemented in the clinical settings against the devastating consequences of ischemic heart disease. Over the last 10 years, our laboratory has demonstrated that phosphodiesterase-5 (PDE-5) inhibitors, including sildenafil (Viagra®), vardenafil (Levitra®) and tadalafil (Cialis®), which are widely prescribed drugs to treat male erectile dysfunction, can induce powerful cardioprotective effect against ischemia-reperfusion (I-R) injury [1], [2] and AMI-induced heart failure [3] in rabbits and mice. We also identified a signaling cascade that involves both endothelial and inducible nitric oxide synthases (eNOS and iNOS) that promote generation of NO [2], [4], enhanced formation of cGMP and activation of protein kinase G (PKG) [5] that result in cardioprotection.

Histone deacetylases (HDACs) have been shown to regulate cardiac hypertrophy and other cardiac diseases [6]. Among three recognized classes of HDACs, class I and class II are zinc-dependent amidohydrolases, whereas class III is nicotinamide adenine dinucleotide (NAD^+^)-dependent, which is termed as sirtuins due to their homology with the yeast HDAC - Sir2. SIRT1 (sirtuin 1 - silent mating type information regulation 2 homolog 1) is part of the class III sirtuin family of HDACs and it catalyzes a reaction in which nicotinamide is liberated from NAD^+^ and the acetyl group of the substrate is transferred to cleaved NAD^+^, generating the novel metabolite O-acetyl-ADP ribose [7]. Since SIRT1 can deacetylate a variety of substrates, it is involved in a broad range of physiological functions, including control of gene expression, cell cycle regulation, apoptosis, DNA repair, metabolism, oxidative stress response and aging [8]. Mice deficient in Sir2 (the murine homolog of SIRT1) exhibited developmental abnormality in the heart and rarely survive postnatally, suggesting that SIRT1 has important function in the heart [9]. SIRT1 activity has also been correlated with metabolic state of cells and it is believed to be nutrient regulated due in part to its dependency on NAD [10], [11]. SIRT1 is also an important regulator of cell defenses and survival in response to stress [10], [11]. For example, SIRT1 provides protection against apoptosis and plays an essential role in mediating the survival of cardiomyocytes under stress *in vitro* [9]. Conversely, reduced SIRT1 activity in cardiomyocytes contributes to myocyte cell death during heart failure [12]. More recently, SIRT1 has been linked to the anti-ischemic cardioprotection by 6-month caloric restriction [13], ischemic preconditioning [14], [15], or resveratrol (RSV) [16]–[18], the best known small molecule activator of SIRT1, which allosterically binds to SIRT1 and lowers the Michaelis constant of SIRT1 for both the acetylated substrate and NAD^+^ [10], [11].

The aim of this study was to determine if SIL induces protection against I-R through activation of SIRT1. Based on the reported NO-dependence of SIRT1 induction and cardioprotection by caloric restriction [13], [19], we hypothesized that SIRT1 activation may be necessary for the cardioprotective effect of SIL, which is also mediated by NO signaling [2], [4]. We tested this hypothesis in an *in vivo* murine model of regional myocardial I-R, and compared with RSV, which served as a positive control for SIRT1 activation. In addition, sirtinol, a putative inhibitor of SIRT1’s HDAC activity [10], [11] was used to determine the causative role of SIRT1 in SIL-induced late cardioprotection. Preliminary results of the present study were presented at the 83^rd^ Scientific Sessions of the American Heart Association held at Chicago, U.S.A. in November 2010 [20].

## Materials and Methods

### Animals

Adult male ICR mice weighing 35.5±5 g were supplied by Harlan Sprague Dawley Co. (Indianapolis, IN). All animal experiments were conducted under the guidelines on humane use and care of laboratory animals for biomedical research published by the U.S. National Institutes of Health (NIH Publication No. 85–23, revised 1996). All experimental preparations and protocols involving animals were reviewed and approved by the Animal Care and Use Committee of Virginia Commonwealth University.

### Drugs and Preparation

Resveratrol powder was purchased from Sigma-Aldrich (St. Louis, MO; item# R5010) and was dissolved in 15% dimethyl sulfoxide (DMSO) and saline (0.9% NaCl containing 2.5 mM CaCl_2_). Sildenafil citrate powder was kindly provided by Pfizer and dissolved in saline. Sirtinol (Sigma-Aldrich; item# S7942) was dissolved in 10% warm PBS and sonicated until homogenous.

### Drug Treatment Regimen and in vivo Myocardial Ischemia-reperfusion Protocol

**Figure 1:**
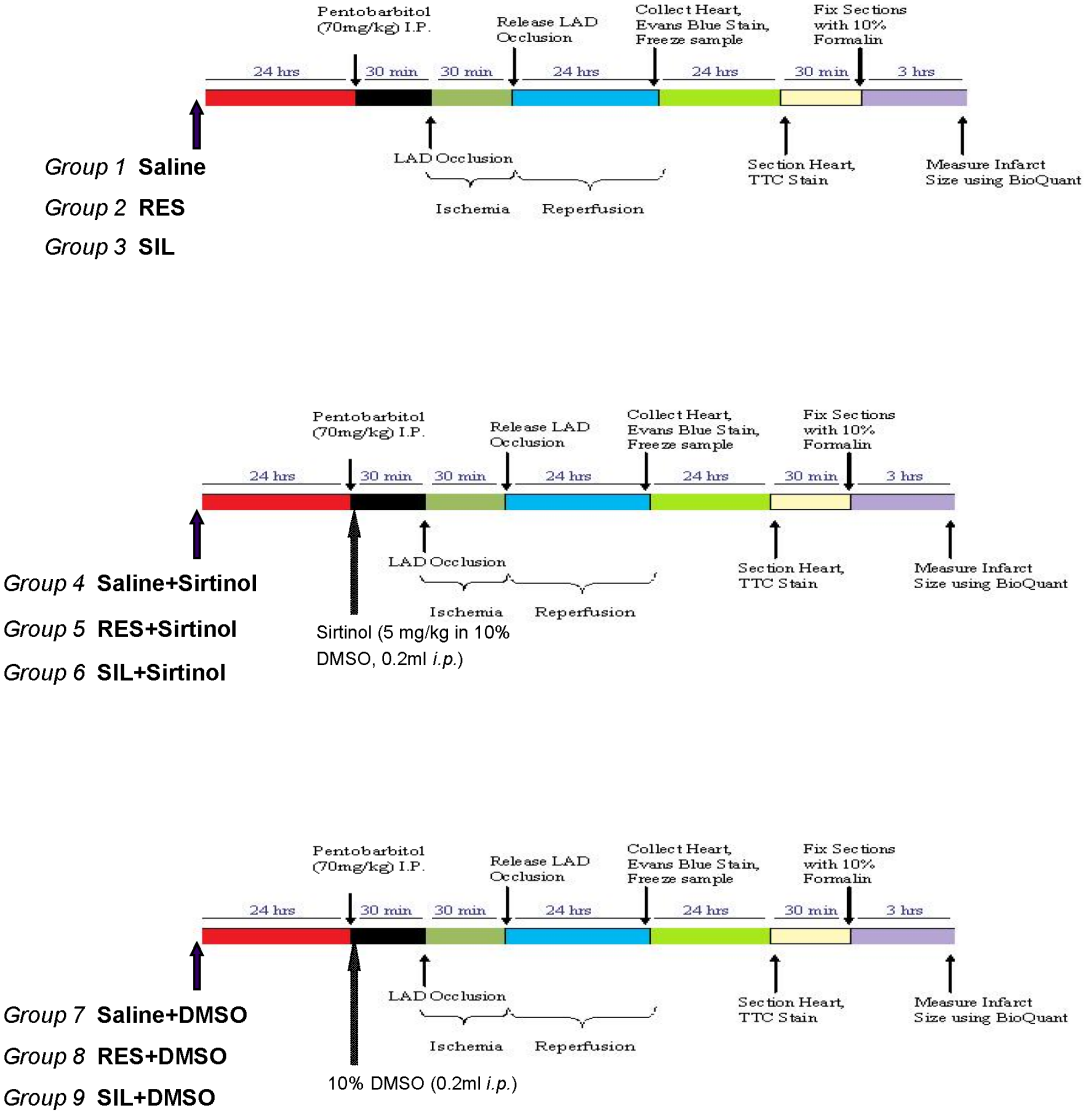
Illustrated description of the experimental protocols using an *in vivo* model of myocardial infarction induced by 30 min of regional ischemia and 24 hours of reperfusion. Note that the drug pretreatments via *i.p.* injection were carried out 24 hours prior to the onset of ischemia at the following dosage: Saline (0.2 ml, served as the Control group); RES (Resveratrol, 5 mg/kg); SIL (Sildenafil, 0.7 mg/kg).

As illustrated in Fig. 1, SIL (0.7 mg/kg), RSV (5 mg/kg), or volume-matched saline (0.2 mL) was injected intraperitoneally (*i.p.*) to the mice, 24 hours prior to I-R. In the SIRT1 inhibitor studies, either sirtinol (5 mg/kg in 10% DMSO) or 10% DMSO (0.2 ml) was given *i.p.* 30 min prior to I-R. The cardioprotective dose of SIL was chosen based on our previous studies [1]–[3]. The *in vivo* I-R surgery procedures were performed by a surgeon who was blinded without knowing the type of drug administered to the individual mice. The methodological details were identical to our previous report [21]. The anesthetized (pentobarbital sodium, 70 mg/kg, *i.p.*), intubated, and mechanically ventilated mouse underwent ligation of left anterior descending coronary artery for 30 min followed by 24 hours of reperfusion until myocardial infarct size was determined.

### Measurement of Infarct Size and Area at Risk

At the end of reperfusion, the mouse was re-anesthetized with pentobarbital sodium and the heart was excised and mounted onto a Langendorff apparatus for washing out blood with saline, and then infused with 2 mL of 10% Evans blue dye and stored at −20°C. The frozen heart was cut into six to eight transverse slices, which were stained with 10% triphenyl tetrazolium chloride solution for 30 min at room temperature. The infarct area and area at risk were measured using computer morphometry (Bioquant 98) as described previously [21]. The risk area was calculated as total ventricular area minus the area of cavities. The infarct size was presented as percentage of the risk area.

### Heart Tissue Sample Collection, Homogenization, Protein Extraction and Purification for Measurement of SIRT1 Activity

Following the above-described treatments, the hearts were harvested under pentobarbital anesthesia and stored at −80°C until further use. A subgroup of SIL, RSV, or saline-treated mice was subjected to I-R 24 hours after the drug treatment. The SIRT1 inhibitor - sirtinol or DMSO was administered 30 min prior to I-R. At the end of I-R protocol, the heart samples were collected and stored as described above. The frozen hearts were then ground with a mortar and pestle in liquid nitrogen. The tissues were homogenized mechanically in a lysis buffer (without protease inhibitors), containing 10 mM Tris-HCl (pH 7.4), NP-40 0.5%, 250 mM sucrose, 0.1 mM EGTA, 10 mM NaCl, 15 mM MgCl_2_, 1 mM PMSF, 1 mM Na_3_VO_4_, and 1 mM NaF. The tissue homogenates were spun through 4 mL of sucrose 30%, 10 mM Tris HCl (pH 7.5), 10 mM NaCl, and 3 mM MgCl_2_ at 1,300×*g* for 10 min at 4°C. The pellet was washed with cold 10 mM Tris-HCl (pH 7.5) and 10 mM NaCl. The nuclei were suspended in 100 µL of extraction buffer containing 50 mM HEPES KOH (pH 7.5), 420 mM NaCl, 0.5 mM EDTA Na_2_, 0.1 mM EGTA, and glycerol 10%, sonicated for 30 s, and stood on ice for 30 min. After centrifugation at 13,000 rpm for 10 min, an aliquot of the supernatant (crude extract nuclear) was used to determine protein concentration using a Bio-Rad assay.

Subsequently, all of the protein samples extracted from the heart tissues or isolated cardiomyocytes were immunoprecipitated with SIRT1 antibody according to the manufacturers’ instruction. In brief, 1 µg of SIRT1 primary antibody (Cyclex, Nagano, Japan) was incubated with 250 µg of protein in extraction buffer overnight at 4°C. Protein A agarose beads were then incubated with the mixture overnight at 4°C. The mixture was then centrifuged for 30 sec. The pellet was washed three times with 250 µL of 1× cell lysis buffer (Cyclex) and 250 µL of Sir2 assay buffer (50 mM Tris-HCl, pH 8.8, 4 mM MgCl_2_, 0.5 mM DTT). The pellet was then suspended in 100 µL of Sir2 assay buffer and kept at −20°C until the measurement of SIRT1 activity.

### Isolation of Ventricular Cardiomyocytes and Cell Collection

The ventricular cardiomyocytes were isolated using an enzymatic technique identical to our previously reported method [4]. The freshly isolated cardiomyocytes were then suspended in minimal essential medium (Sigma-Aldrich, item# M1018, pH 7.35–7.45), containing 1.2 mM Ca^2+^, 12 mM NaHCO_3_, 2.5% fetal bovine serum, and 1% penicillin-streptomycin. The cells were then plated onto 35-mm cell culture dishes that were precoated with 20 µg/mL mouse laminin in phosphate-buffered saline with 1% penicillin-streptomycin for 1 hour. After 1 hour of plating, the cardiomyocytes were incubated with 1 µM SIL or RSV, or without drug treatment (control). Following the incubation, the cells were scraped from the plate and frozen at −80°C until further use for SIRT1 activity analysis.

### SIRT1 Deacetylase Activity Assay

SIRT1 deacetylase activity was evaluated in the whole heart lysate as well as cardiomyocyte lysate according to the previously reported methods [22]. A deacetylase fluorometric assay kit (Sir2 Assay Kit, Cyclex) was used. The final reaction mixture (100 µL) contained: 50 mM Tris-HCl (pH 8.8), 4 mM MgCl_2_, 0.5 mM DTT, 0.25 mA/mL Lysyl endopeptidase, 1 µM trichostatin A, 200 µM NAD, and 10 µL of the crude extract nuclear sample. The fluorescence intensity at 440 nm wavelength (exc. 340 nm) was measured every 5 min for a total of 30 min immediately after the addition of fluorosubstrate peptide, and then right after the addition of stop solution. 60 µM of sirtinol - a SIRT1 inhibitor and trichostatin A - an inhibitor of class I and II HDACs [9] were also used. All measurements were performed in duplicate and the results are reported as arbitrary units of relative fluorescence.

### Western Blot Analysis for SIRT1 Expression

The murine hearts were homogenized and protein expression was analyzed using techniques we previously reported [5]. In brief, the heart tissues were ground with a mortar and pestle under liquid nitrogen into fine powder. Total soluble protein was extracted from the heart lysates with RIPA buffer plus PMSF, protease inhibitors, Na_3_VO_4_, and β-mercaptoethanol. A mechanical homogenizer was used to homogenize the samples, which were subsequently sonicated. The homogenate was then centrifuged at 14,000×*g* for 15 min at 4°C and the supernatant was recovered and stored at −80^o^C until used. Following quantification of the sample protein concentration using a Bio-Rad assay, 100 µg of protein from each sample was separated by SDS-PAGE on a 7.5% polyacrylamide gel, transferred onto nitrocellulose membrane and then blocked with 5% nonfat dry milk in TBST (10 mM Tris-HCl, pH 7.4, 100 mM NaCl, and 0.1% Tween 20) for 1 h. The membrane was incubated with a primary antibody (at 1∶1000 dilution) for SIRT1 (Sigma-Aldrich) or β-actin (Santa Cruz Biotechnology) overnight at 4°C. The membrane was then washed with TBST and incubated with secondary antibody (1∶2000 dilution, 1 h at room temperature). The blots were developed using a chemiluminescent system (ECL Plus; Amersham Biosciences). Densitometry was used to quantify the optical density of SIRT1 for each band and normalized with the intensity of β-actin, presented as ratio of SIRT1/β-actin [4].

### Data Analysis and Statistics

Data are presented as mean ± S.E. The differences between groups were analyzed with one-way analysis of variance followed by Student-Newman-Keuls post hoc test for pair-wise comparison. P<0.05 was considered to be statistically significant.

## Results

### Effect of SIRT1 Inhibitor on SIL-induced Infarct-limiting Cardioprotection

**Figure 2:**
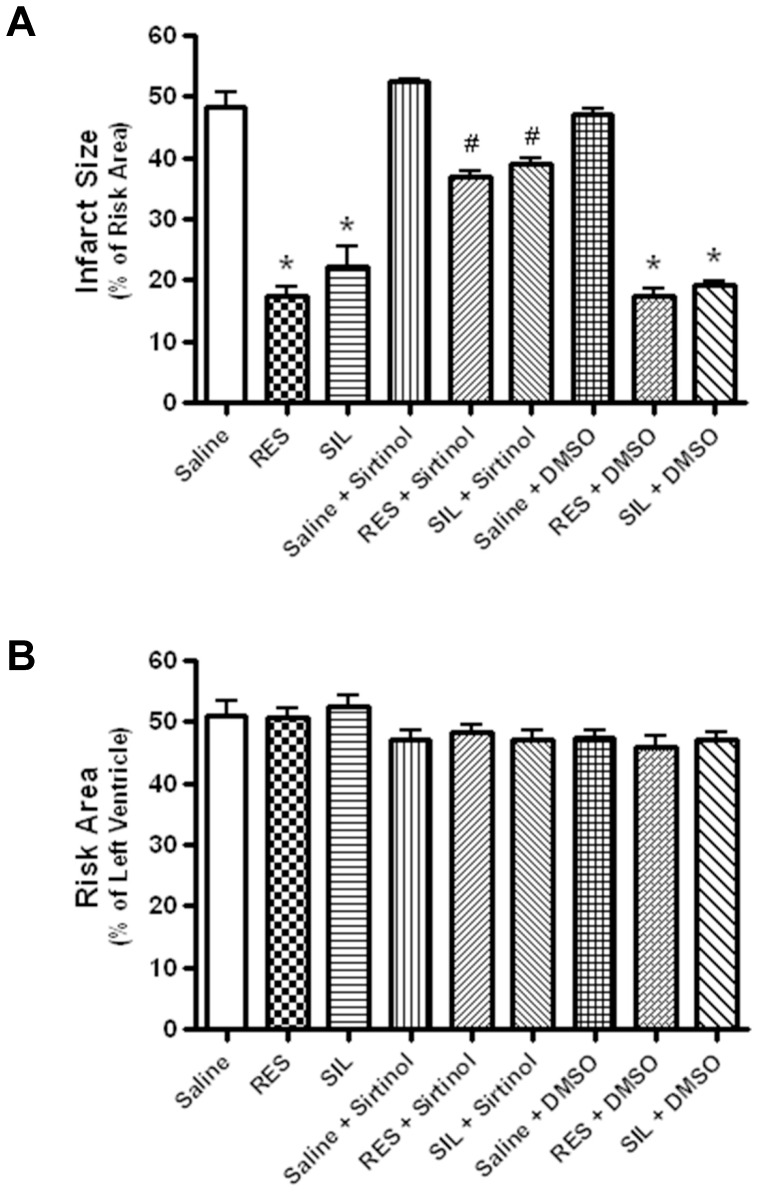
A. Myocardial Infarct Size. Infarct size was significantly (P<0.001 vs. saline controls) reduced in the Sildenafil-treated, Resveratrol-treated, Sildenafil+DMSO-treated, and Resveratrol+DMSO-treated groups. Sirtinol abolished the cardioprotective effects of both Sildenafil and Resveratrol. n = 6/Group, * = P<0.001 vs. Saline Controls, # = P<0.05 vs. other groups. B. Area at Risk**.** The area at risk was presented as percentage of the total left ventricular area that underwent ischemia. n = 6/group, no significant difference was found among all groups.

As shown in Fig. 2A (Mean ± SE, n = 6/group), the saline-treated control mice had large infarct size (48.3±5.9% of the risk area) following 30 min of ischemia and 24 hours of reperfusion. SIL significantly reduced myocardial infarct size to 22.3±7.6% (P<0.001) as compared to the saline group. RSV, a putative activator of SIRT1, also significantly reduced infarct size to 17.4±3.7% (P<0.001). The infarct-limiting cardioprotection by SIL or RSV was abrogated by sirtinol – a SIRT1 inhibitor administered *i.p. *30 min prior to ischemia (Fig. 1A), indicated by the large infarct size in SIL+Sirtinol group (38.9±2.5%) and RSV+Sirtinol group (37.1±1.8%), which were not significantly different from the Saline (48.3±5.9%) or Saline+Sirtinol (52.5±1.2%) groups (P>0.05). 10% DMSO (the solvent of sirtinol) alone had no significant effect on infarct size and it also did not block the protective effects of SIL and RSV, as indicated by the significantly smaller infarct size in the SIL+DMSO and RSV+DMSO groups as compared with Saline+DMSO group (Fig. 2A). All of the drug treatments did not affect the amount of area at risk, as indicated in Fig. 2B.

### Effect of SIL on SIRT1 Deacetylase Activity in Intact Heart and Isolated Cardiomyocytes

**Figure 3:**
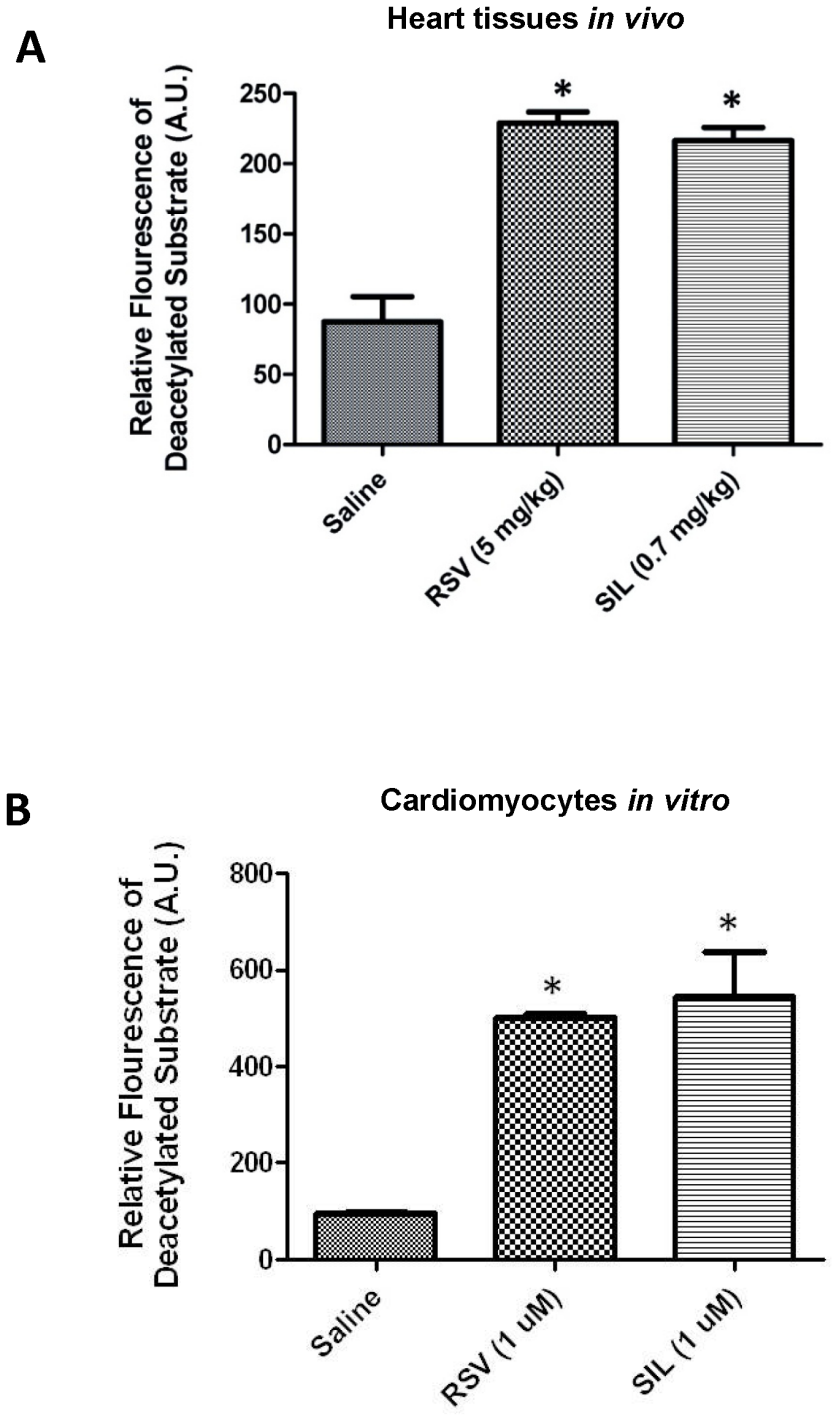
Enhanced SIRT1 deacetylase activity in heart tissues from the mice treated *i.p.* with single dose of resveratrol (RSV, 5 mg/kg) or sildenafil (SIL, 0.7 mg/kg) for 24 hours (Graph A); *P<0.001 vs. Saline (n = 3/group). Graph B shows SIRT1 deacetylase activity in isolated cardiomyocytes following 1(1 µM, n = 3/group) *in vitro*. *P<0.05 vs. the untreated control group.

We measured the relative fluorescence of deacetylated SIRT1 substrate in the homogenized heart samples following various drug treatment regimens. As shown in Fig. 3A, SIL (0.7 mg/kg, *i.p.*) or RSV (5 mg/kg, *i.p.*) resulted in a significant increase in cardiac SIRT1 activity 24 hours after the drug pre-treatment, as compared with the Saline controls (P<0.001). The levels of SIRT1 activation by SIL and RSV were largely comparable (*i.e. P*>0.05 SIL versus RSV). Furthermore, we investigated the direct effect of SIL or RSV on SIRT1 activity in ventricular myocytes in an *in vitro* cultured cell model. We observed that 1 hour incubation with 1 µM SIL or RSV led to a significant increase in SIRT1 activity in the isolated cardiomyocytes (*P*<0.05; Fig. 3B).

**Figure 4:**
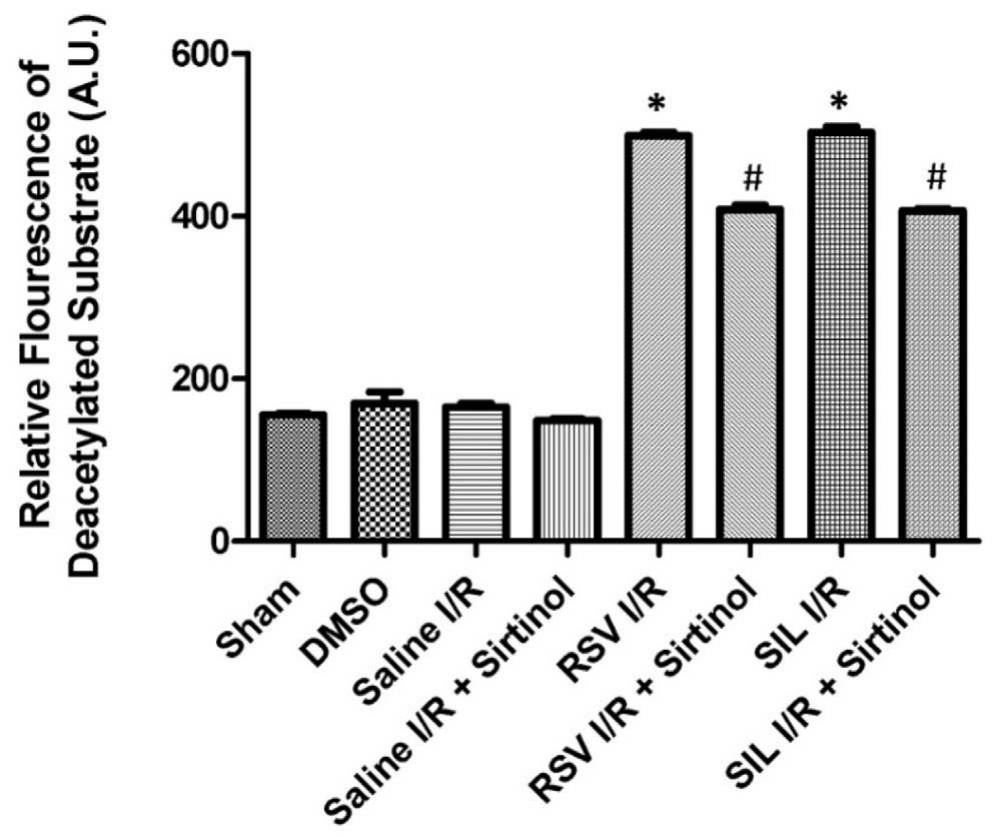
SIRT1 deacetylase activity in the heart tissues from the mice pretreated (*i.p.*) with saline (0.2 mL), RSV (5 mg/kg), and SIL (0.7 mg/kg) 24 hours prior to 30 min of regional ischemia and 24 hours of reperfusion. The SIRT1 inhibitor - sirtinol (5 mg/kg) was administered (*i.p.*) 30 min prior to the onset of ischemia. The Sham group serves as surgical controls, whereas Saline and DMSO groups serve as the drug solvent/vehicle controls. Data are Mean ± SE (n = 3/group). *P<0.0001 vs. Control group. #*P*<0.05 vs. all other groups.

As shown in Fig. 4, SIL or RSV pre-treatment (24 hours prior to I-R) significantly enhanced SIRT1 activity in the post-I-R heart as compared with the Saline-treated controls (P<0.0001). The inhibitory effect of sirtinol on cardiac SIRT1 activities in SIL or RSV-treated mice was also apparent (P<0.05; Fig. 4). I-R alone did not alter cardiac SIRT1 activity.

### Effect of SIL on Cardiac SIRT1 Protein Expression

**Figure 5:**
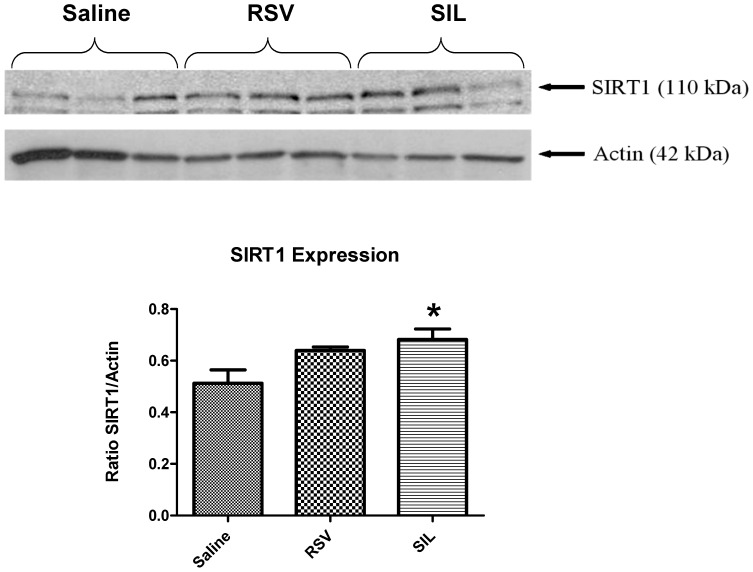
Cardiac expression of SIRT1 in mice pretreated (*i.p.*) with saline (0.2 mL), RSV (5 mg/kg), and SIL (0.7 mg/kg) 24 hours prior to heart tissue collection. *Top:* Western blots showing protein expression of SIRT1 and Actin (as the loading control). *Bottom:* Densitometric quantification of the Western blotting bands. The values of SIRT1 are normalized against Actin for each sample (n = 3/group, *indicates *P*<0.05 vs. Saline).

Western blot analysis shows increase in cardiac SIRT1 protein expression 24 hours following the SIL treatment (Figure 5), which was confirmed by the densitometric results expressed as SIRT1/β-actin ratio (*P*<0.05, SIL group *versus* Saline group, Figure 5 lower panel).

## Discussion

The class III HDAC family member SIRT1 has been shown to protect against cardiovascular diseases, cancer, and neurodysfunction [10]. Considering its functional importance in the control of gene expression, cell cycle regulation, apoptosis, DNA repair, metabolism, oxidative stress response and aging [8], identification of new pharmacological interventions to regulate SIRT1 activity for therapeutic interventions in human disorders has been a focal point of intensive research. Our results provide the first evidence which shows that cardioprotective dose of SIL - a selective PDE-5 inhibitor enhances SIRT1 activity in the heart (Fig. 3). Furthermore, our data shows that SIRT1 activation plays a causative role in SIL-induced late cardioprotection, because SIRT1 inhibitor – sirtinol completely blocked the infarct-limiting effect of SIL (Fig. 2A). It is notable that the SIRT1 activation or upregulation by SIL pretreatment was observed in unstressed (*i.e.*, non-ischemic) (Fig. 3A, Fig. 5) as well as in hearts following I-R injury (Fig. 4).

We further investigated if SIL could directly augment SIRT1 activity in the isolated mouse cardiomyocytes *in vitro.* Our results show that incubation with 1 µM SIL (an established cytoprotective dose in our previous publications [4], [5] in the culture medium (37°C, 5% CO_2_) for 1 hour significantly activated SIRT1 in the isolated cardiomyocytes (Fig. 3B). These results suggest that SIL rapidly activates SIRT1 in cardiomyocytes and mediates protection during a wide range of time window, *i.e.* 24 hours. An interesting observation in the present study is that the cardioprotective dose of SIL activated SIRT1 similar to RSV, a putative activator of SIRT1 [16]–[18]. The magnitude of increase in cardiac SIRT1 activity following treatment with low dose SIL (0.7 mg/kg) was similar to the one induced by 5 mg/kg RSV (Fig. 3A and Fig. 4).

There could be other advantages for SIL considering its well proven safety profiles as a FDA-approved and widely prescribed drug for managing patients with male erectile dysfunction and pulmonary hypertension. Interestingly, the oral administration of RSV attenuated established monocrotaline-induced pulmonary hypertension indices, including right ventricular systolic pressure, right ventricular hypertrophy, and medial thickening of intrapulmonary arteries [23]. Moreover, SIRT1 inhibition augmented proliferation of pulmonary artery smooth muscle cells, as assessed by DNA mass and suppressed atrogin mRNA expression. Thus the SIRT1-activating effects of SIL reported in the present study provides a new and exciting mechanistic explanation to utilize this drug and potentially other PDE-5 inhibitors in reducing ischemic heart tissue damages as well as pulmonary hypertension, considering the essential role played by SIRT1 in regulating cell defenses and survival in response to stress [10], [11]. Nevertheless, further investigations should be conducted to elucidate the signaling network linking PDE-5 inhibition with SIL and SIRT1 activation in the heart, which leads to cardioprotection against I-R injury. In light of our previous studies [2], [4] showing SIL significantly enhances levels of mRNA and/or protein expression of eNOS and iNOS in intact heart and isolated cardiomyocytes, we postulate that this NO signaling may have a significant role in the activation of SIRT1 as previously shown in the calorie restriction models [13], [19]. Moreover, it has been shown that chronic inhibition of NOS blocked the calorie restriction-induced increase in nuclear SIRT1 content as well as ischemic tolerance in the heart [13], [19]. However, the exact signal transduction cascade and the role of protein kinases such as PKG in activating SIRT1 remain to be investigated. It is notable that the effects of SIL on other members of Sir2 family such as SIRT3– a cardiac abundant isoform have not yet been investigated and may represent an additional important target of PDE-5 inhibitors.

## Conclusion

We have demonstrated that SIL-induced late cardioprotection is mediated by SIRT1 activation. This newly identified SIRT1-activating property of SIL may have enormous therapeutic implications and benefits in reducing not only the myocardial I-R injury, but also other types of cardiovascular disorders related to aging and metabolic dysregulation such as Type 2 diabetes.
